# Hypergravity Provokes a Temporary Reduction in CD4+CD8+ Thymocyte Number and a Persistent Decrease in Medullary Thymic Epithelial Cell Frequency in Mice

**DOI:** 10.1371/journal.pone.0141650

**Published:** 2015-10-29

**Authors:** Ryosuke Tateishi, Nobuko Akiyama, Maki Miyauchi, Riko Yoshinaga, Hiroki Sasanuma, Takashi Kudo, Miki Shimbo, Masahiro Shinohara, Koji Obata, Jun-ichiro Inoue, Masaki Shirakawa, Dai Shiba, Hiroshi Asahara, Nobuaki Yoshida, Satoru Takahashi, Hironobu Morita, Taishin Akiyama

**Affiliations:** 1 Division of Cellular and Molecular Biology, The Institute of Medical Science, The University of Tokyo, Tokyo, Japan; 2 Mouse epigenetics project, ISS/Kibo experiment, Japan Aerospace Exploration Agency, JAXA, Tsukuba, Japan; 3 Laboratory of Developmental Genetics, Center for Experimental Medicine and Systems Biology, The University of Tokyo, Tokyo, Japan; 4 Laboratory Animal Resource Center, University of Tsukuba, Tsukuba, Ibaraki, Japan; 5 Department of Anatomy and Embryology, Faculty of Medicine, University of Tsukuba, Tsukuba, Ibaraki, Japan; 6 Department of Systems BioMedicine, Graduate School of Medical and Dental Sciences, Tokyo Medical and Dental University, Tokyo, Japan; 7 JST, PRESTO, Kawaguchi, Saitama, Japan; 8 Department of Physiology, Gifu University Graduate School of Medicine, Gifu, Japan; 9 JEM Utilization Center, Human Spaceflight Technology Directorate, JAXA, Tsukuba, Ibaraki, Japan; Keio University School of Medicine, JAPAN

## Abstract

Gravity change affects many immunological systems. We investigated the effects of hypergravity (2G) on murine thymic cells. Exposure of mice to 2G for three days reduced the frequency of CD4^+^CD8^+^ thymocytes (DP) and mature medullary thymic epithelial cells (mTECs), accompanied by an increment of keratin-5 and keratin-8 double-positive (K5^+^K8^+^) TECs that reportedly contain TEC progenitors. Whereas the reduction of DP was recovered by a 14-day exposure to 2G, the reduction of mature mTECs and the increment of K5^+^K8^+^ TEC persisted. Interestingly, a surgical lesion of the inner ear’s vestibular apparatus inhibited these hypergravity effects. Quantitative PCR analysis revealed that the gene expression of Aire and RANK that are critical for mTEC function and development were up-regulated by the 3-day exposure and subsequently down-regulated by the 14-day exposure to 2G. Unexpectedly, this dynamic change in mTEC gene expression was independent of the vestibular apparatus. Overall, data suggest that 2G causes a temporary reduction of DP and a persistent reduction of mature mTECs in a vestibular system-dependent manner, and also dysregulates mTEC gene expression without involving the vestibular system. These data might provide insight on the impact of gravity change on thymic functions during spaceflight and living.

## Introduction

Spaceflight affects various immune systems [1–3]. Altered gravity is one of the hostile conditions during spaceflight, specifically microgravity in orbit and high gravity force at launch and landing. Several studies on spaceflight missions suggested the impact of gravity change on immune systems [2, 3]. Due to the cost and limited availability of spaceflight missions, some ground-based models using experimental animals also have been developed to test the effect of gravity changes on physiological parameters [4–7]. Hindlimb unloading model [4, 6] and chronic centrifugation model [8–11] for rodents have been used to study the effects of microgravity and hypergravity on immune systems, respectively. Together with spaceflight studies, these ground studies have revealed the deleterious effect of gravity change on many immunological parameters.

The mechanism by which altered gravity dysregulates immune systems remains elusive. In addition to the direct effects on immune cells [3], gravity sensing through other physiological systems might indirectly disturb immune homeostasis [3]. For instance, otolith organs in the vestibular apparatus of the inner ear detect linear acceleration, thereby sensing gravitational force. This sensing of gravity change by the vestibular apparatus may eventually affect immune systems via autonomic nervous systems. Moreover, altered gravity causes changes in the musculoskeletal system [12] and shift of body fluid [13]. The link between these sensing systems and immune dysregulation in altered gravity has yet to be elucidated.

The thymus is a primary lymphoid organ supporting the differentiation of T cells [14]. In the thymus, immature thymic T cells (thymocytes) interact with thymic epithelial cells (TECs) [15, 16]. Such interactions are required for the differentiation and selection of self-MHC restricted and self-tolerant T cells [15, 16]. TECs are subdivided into cortical TECs (cTECs) and medullary TECs (mTECs), which are differentiated from common progenitors [15]. cTECs play essential roles in early thymocyte development and the positive selection of self-MHC-restricted T cells. On the other hand, mTECs ectopically express self-tissue specific antigens under the control of a nuclear protein autoimmune regulator (Aire) in which mutation causes autoimmune disease [17, 18]. The direct or indirect presentation of these self-antigens by mTECs is critical for eliminating self-antigen reactive T cells and generating immunosuppressive regulatory T cells [17, 18], thereby preventing the onset of autoimmunity in the body. Moreover, recent studies suggested that mTECs control tumor immunity by selecting tumor-reactive T cells and regulatory T cells [19–21].

Many extrinsic factors such as pathological condition, psychological and physiological stresses, and therapeutic interventions (e.g. irradiation or chemotherapy) cause reductions in thymic weight and numbers of thymocytes and TECs [22–24]. The effect of spaceflight on thymic weight has been studied in rodents [25–28]. Spaceflight has reportedly decreased thymic weight [25–27] or left it unchanged [28]. The different outcomes may be due to the schedule of sample preparations and species of rodent used in these experiments [28]. Hindlimb unloading also caused a reduction of thymic weight [29]. With regard to the effects of hypergravity, an earlier study showed that a chronic centrifuge did not change thymic weight in rats [9]. Subsequent studies revealed that centrifuge-induced hypergravity resulted in a reduction of thymic weight [10, 11]. In one such study [10], although the hypergravity-dependent reduction of relative thymic weight was significant at an early time point, the reduction was recovered in mice treated with relatively long-term exposure to hypergravity. Although the effects of gravity change on thymic weight and total thymocytes have been reported relatively well, few studies have determined the types of thymocytes impaired by changing gravity. Moreover, the effect of gravity change on TECs has yet to be reported.

In this study, we investigated the effects of hypergravity (2G) induced by chronic centrifugation on the frequency of thymocytes and TECs in mice. We describe that hypergravity causes a reduction in the number of CD4^+^CD8^+^ thymocytes and mature mTECs, accompanied by an increment in TECs expressing both keratin-5 and keratin-8 (K5^+^K8^+^) that reportedly contain common TEC progenitors and mTEC progenitor [30–32]. Whereas the reduction of CD4^+^CD8^+^ thymocytes was temporary, mature mTECs were persistently decreased by hypergravity exposure. Importantly, a surgical lesion of the inner ear’s vestibular apparatus remarkably alleviated these changes. We also reported that 2G exposure resulted in the dysregulation of gene expression in mTECs, independent of the vestibular apparatus.

## Results

### Short-term exposure of mice to hypergravity causes a reduction of CD4^+^CD8^+^ thymocytes in vestibular apparatus-dependent manner

**Fig 1:**
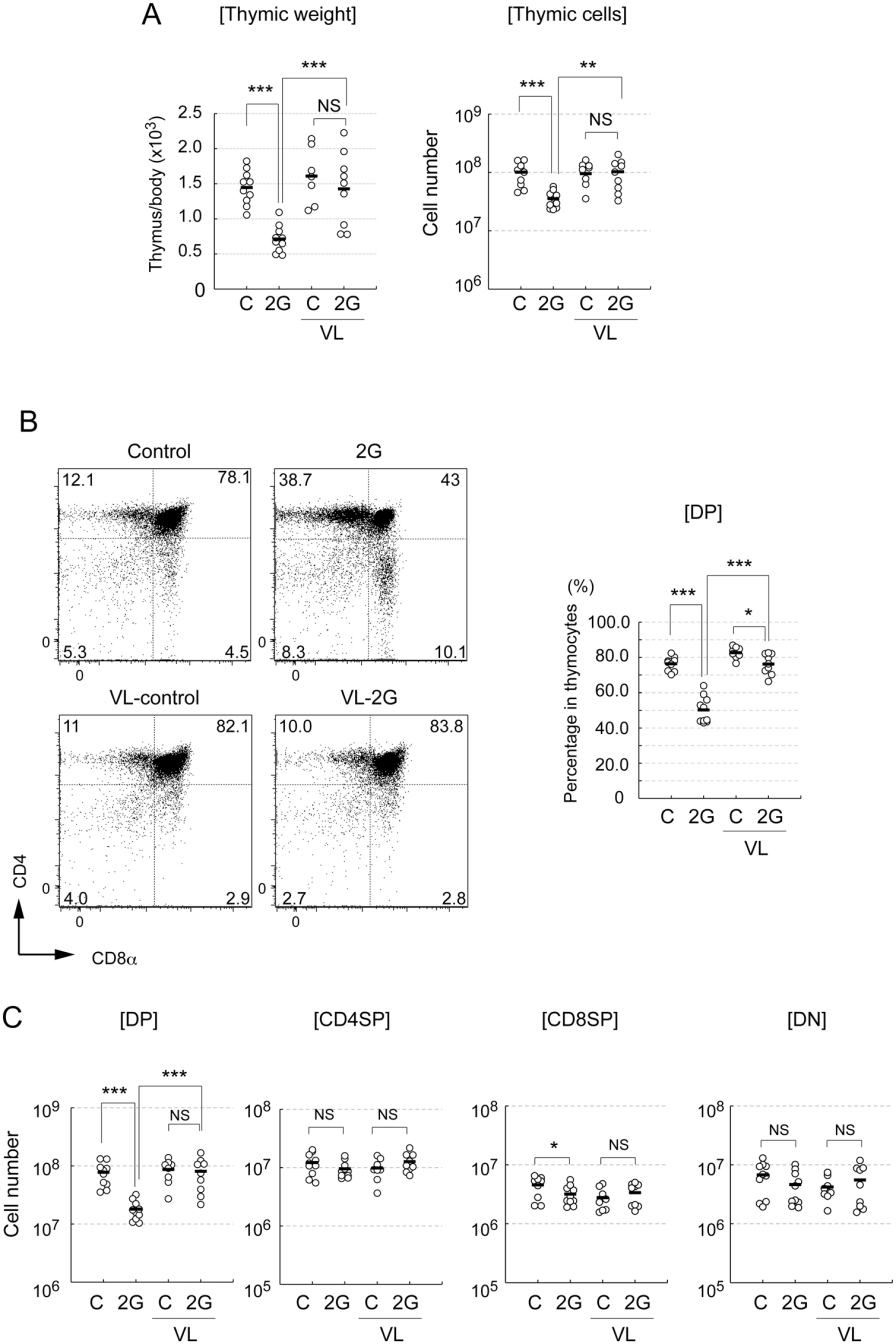
The short-term hypergravity exposure causes a reduction of CD4^+^CD8^+^ double positive thymocytes in a vestibular apparatus-dependent manner. (A) Ratio of thymic weight to body weight (left) and total thymic cell number (right) of mice exposed to 2 *g* gravity (labeled as 2G) for 3 day or normal gravity control (labeled as C). Vestibular apparatus were surgically disrupted in some groups of mice (labeled as VL). N = 10 for C and 2G, N = 8 for C with VL, and N = 9 for 2G with VL. The asterisks indicate statistical significance at ***P < 0.001 and **P < 0.01 (Student’s *t*-test). NS indicates that the difference is not significant (Student’s *t*-test). (B) Flow cytometric analysis of thymocytes in mice exposed to 2G for 3 days. Thymocytes were analyzed by staining with CD4 and CD8α antibodies (left figures). Numbers in panels indicates percentage of each fraction. Percentages of CD4- and CD8-double positive thymocytes (CD4^+^CD8^+^DP) in total thymocytes are summarized in right figures. Mice were exposed to 2 *g* gravity (2G) for 3 days. “C” indicates 1G control. Vestibular apparatus are surgically disrupted in some groups of mice (VL). N = 10 for C and 2G, N = 8 for C with VL, and N = 9 for 2G with VL. The asterisks indicate statistical significance at ***P < 0.001 and *P < 0.05 (Student’s *t*-test). NS indicates that the difference is not significant (Student’s *t*-test). (C) Cell numbers of CD4^+^CD8^+^ (CD4^+^CD8^+^DP), CD4^+^CD8^-^ (CD4SP), CD4^-^CD8^+^ (CD8SP), and CD4^-^CD8^-^ (DN) fractions in mice exposed to 2G for 3days. N = 10 for C and 2G, N = 8 for C with VL, and N = 9 for 2G with VL. The asterisks indicate statistical significance at ***P < 0.001 and *P < 0.05 (Student’s *t*-test). NS indicates that the difference is not significant (Student’s *t*-test).

Mice were exposed to 2G by chronic centrifugation. Exposure to 2G for 3 days significantly resulted in a reduction of thymic weight and the number of cells as compared to the control 1G mice (Fig 1A), which is consistent with previous studies [10, 11]. To investigate the role of the vestibular apparatus in 2G-dependent effects, we performed surgery for lesion of the inner ear’s vestibular apparatus (VL) in some mice [33]. Impressively, thymic involution due to 2G exposure was remarkably alleviated in VL mice (Fig 1A). This suggests that sensing gravity change by the vestibular apparatus triggers 2G-depenent thymic involution.

We then investigated the types of cells reduced by short-term exposure to 2G. Thymic T cells were largely separated into four fractions by the expression of CD4 and CD8. Early T cell progenitors initially differentiate into CD4^–^CD8^–^ (DN) cells and then into CD4^+^CD8^+^ thymocytes (DP) in the thymus [14]. DP is then converted to mature T cells expressing either CD4 or CD8 (CD4SP or CD8SP) [14]. We conducted flow cytometric analysis to determine the cell types influenced by 2G exposure. The percentage and cell numbers of DP were significantly reduced by the exposure to 2G (Fig 1B and 1C). VL remarkably alleviated the 2G-dependent reduction in DP frequency (Fig 1B and 1C). On the other hand, percentage of CD4SP, CD8SP, and DN were increased (S1 Fig), which was most likely due to the reduced percentage of DP. Indeed, cell numbers of CD4SP and DN were not practically changed and CD8SP cell number was rather slightly decreased by the 2G exposure (Fig 1C).

Our data suggest that short-term 2G exposure causes a selective reduction of DP by sensing gravity change through the vestibular apparatus. As the development and maintenance of thymocytes are dependent on thymic epithelial cells (TECs), we further addressed the effects of hypergravitiy on the maintenance of TECs.

### Short-term exposure of mice to hypergravity causes a reduction of mature mTECs and an increment in K5^+^K8^+^ TECs in vestibular apparatus-dependent manner

**Fig 2:**
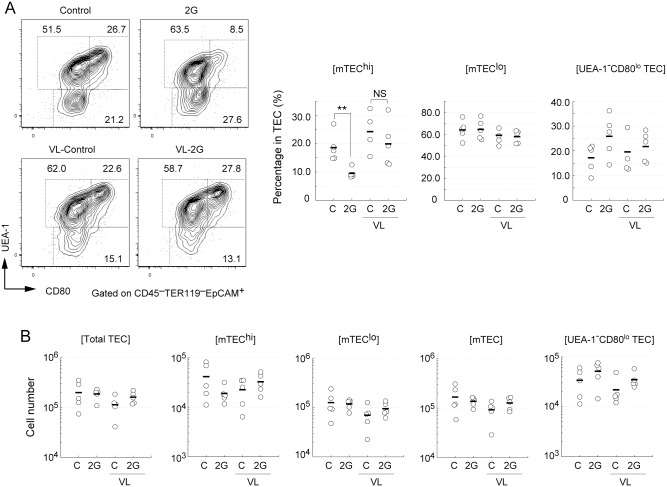
The short-term hypergravity exposure causes a reduction in frequency of mature medullary thymic epithelial cells in a vestibular apparatus-dependent manner. (A) Flow cytometric analysis of thymic epithelial cells (TECs) in mice exposed to 2G for 3 days. Mice were exposed to 2 *g* gravity (2G) for 3 days or left under 1G (Control). Vestibular apparatus are surgically disrupted in some mice (labeled as VL). TECs (CD45^–^TER119^–^ EpCAM^+^) in total thymic cells were analyzed by staining with UEA-1-lectin, an mTEC marker, and CD80 antibody. Numbers in panels indicates percentage of each fraction. The percentages of UEA-1^–^ CD80^lo^ (containing cTECs), UEA-1^+^CD80^high^ (mTEC^hi^), and UEA-1^+^CD80^low^ (mTEC^lo^) cells among thymic stroma cells in the thymus are summarized in right figures. “C” in graphs indicates 1G control. N = 5 each C, 2G, C with VL, and 2G with VL groups. The asterisks indicate statistical significance at **P < 0.01 (Student’s *t*-test). (B) Cell numbers of total TECs (CD45^–^TER119^–^ EpCAM^+^), UEA-1^–^ CD80^lo^ TECs (containing cTECs), mTECs (UEA-1^+^), mTEC^hi^ (UEA-1^+^CD80^high^), and mTEC^lo^ (UEA-1^+^CD80^low^) in the thymus are summarized in figures. N = 5 each C, 2G, C with VL, and 2G with VL groups.

We performed flow cytometric analysis of TECs in mice exposed to 2G for 3 days. We mainly focused on the mTEC fraction because mature mTECs were reportedly sensitive to such stressors as treatment using immunosuppressive drugs [34]. A medullary TEC (mTEC) marker (UEA-1-lectin) and a mature TEC marker (CD80) were used for staining to separate the TEC fraction (CD45^-^TER119^-^EpCAM^+^ cells) in all thymic cells. UEA-1^+^CD80^hi^TECs (mTEC^hi^) were considered mature mTECs, and the UEA-1^+^CD80^lo^TECs (mTEC^lo^) fraction contains immature mTECs [17]. UEA-1-negative fraction (referred to hereafter as UEA-1^–^CD80^lo^ TECs) mainly contains cortical TECs and TEC precursors [32, 35]. The percentage of mTEC^hi^ was decreased by half in the thymus of mice exposed to 2G for three days (Fig 2A and S2A Fig). The average cell number of mTEC^hi^ was also consistently reduced by approximately 50% in the 2G-treated mice although this reduction did not reach statistical significance (Fig 2B and S2B Fig). The 2G-dependent reduction of mTEC^hi^ was minimum in VL mice (Fig 2 and S2 Fig), suggesting that gravity sensing by the vestibular apparatus is responsible for the 2G-dpenendent reduction of mTEC^hi^. In contrast to mTEC^hi^, the frequencies and cell numbers of mTEC^lo^ and UEA-1^–^CD80^lo^ TEC fractions were not significantly influenced by 2G-exposure (Fig 2 and S2 Fig).

**Fig 3:**
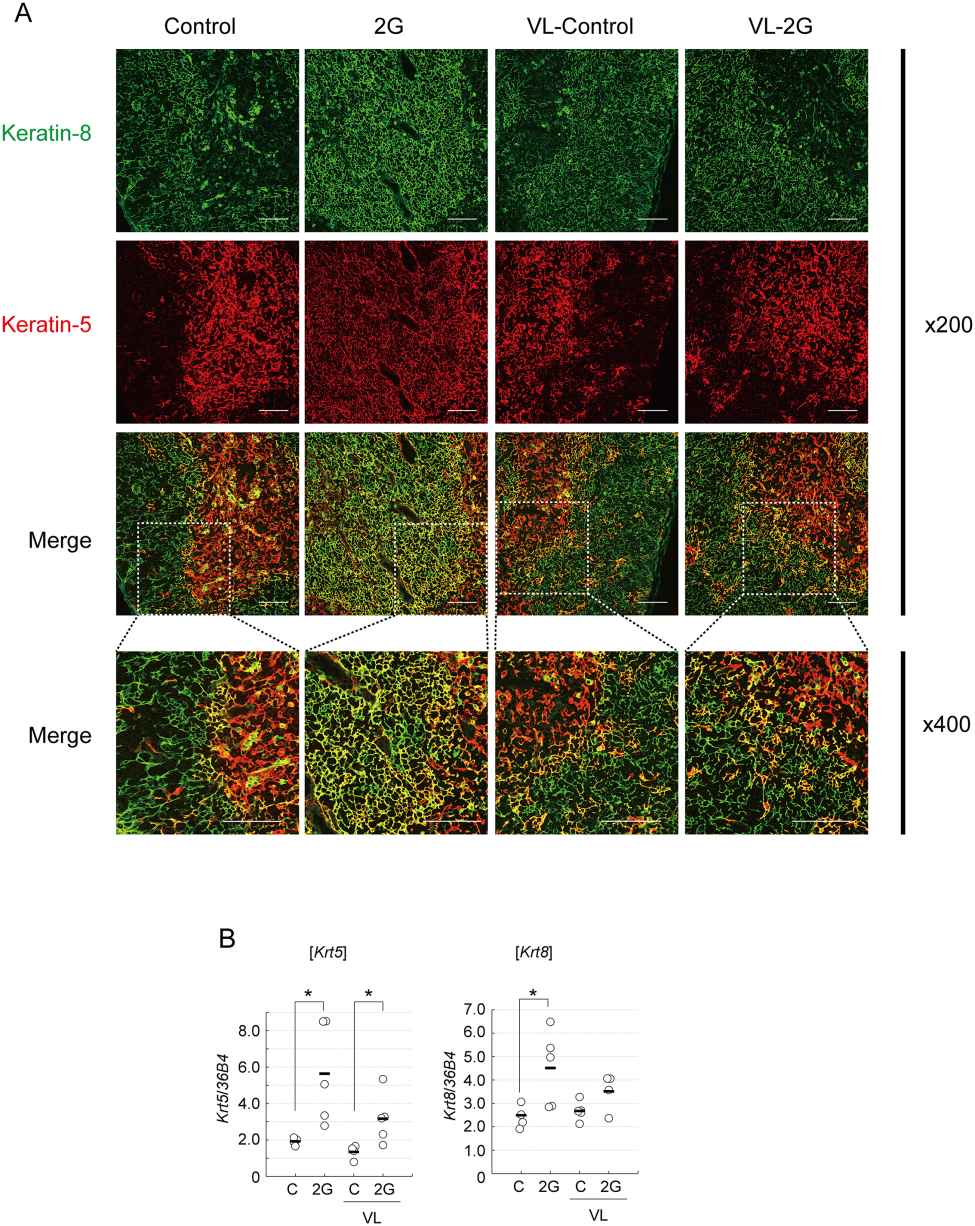
The short-term hypergravity exposure causes an increment of keratin-5 keratin-8-double positive cells in a vestibular apparatus-dependent manner. (A) Immunostaining of thymic section from mice exposed to 2G for 3 days. Thymic frozen sections were immunostained with a combination of anti-keratin-8 antibodies (upper panels) and anti-keratin-5 (upper middle panels). Merged images are shown in lower middle panels. Magnified images (indicated as x40) of white dot rectangles are shown in lower panels. Mice were exposed to 2 *g* gravity (2G) for 3 days or left under 1G (Control). Vestibular apparatus are surgically disrupted in some groups of mice (VL). Data are representatives of 6 independent mice samples (N = 6). Scale bars indicate 100 μm. (B) Expression of keratin-5 and keratin-8 mRNA in the total thymus from mice exposed to 2G for 3 days. Expression of keratin-5 (*Krt5*) and keratin-8 (*Krt8*) mRNAs was evaluated by qPCR analysis. N = 5 each C, 2G, C with VL, and 2G with VL groups. The asterisks indicate statistical significance at *P < 0.05 (Student’s *t*-test).

We further evaluated the effects of 2G exposure on TECs by immunohistochemical staining. Expression of keratin-5 (K5) and keratin-8 (K8) are known to mark heterogeneous populations of TECs [15, 32]. Immunostaining of the thymic sections indicated that the 3-day 2G exposure results in an increment of TECs expressing both K5 and K8 (K5^+^K8^+^) (Fig 3A). K5^+^K8^+^ TECs reportedly contain TEC progenitors [30, 31] and also may represent mTEC precursor populations [32]. Previous studies showed that irradiation and steroid treatment caused increment of K5^+^K8^+^ TECs [36]. Thus, 2G-exposure resulted in a similar outcome with these stressors. VL notably inhibited the increment of K5^+^K8^+^ cells induced by 2G exposure (Fig 3A). qPCR analysis suggested that expression of K5 and K8 mRNAs was increased in the thymus of the 2G-treated mice (Fig 3B). The significant increment of K8 mRNA expression was not found in VL mice, although K5 mRNA was slightly increased even in VL mice (Fig 3B).

Overall, our data suggested that 2G exposure for 3 days provokes a reduction of mTEC^hi^ frequency and an increment in K5^+^K8^+^ TECs. As previous studies have suggested that a reduction of thymic weight by 2G exposure was recovered during prolonged exposure [10], we further investigated the impact of longer 2G exposure on the maintenance of thymocytes and TECs.

### Hypergravity-induced reduction of CD4^+^CD8^+^ thymocytes is temporary

**Fig 4:**
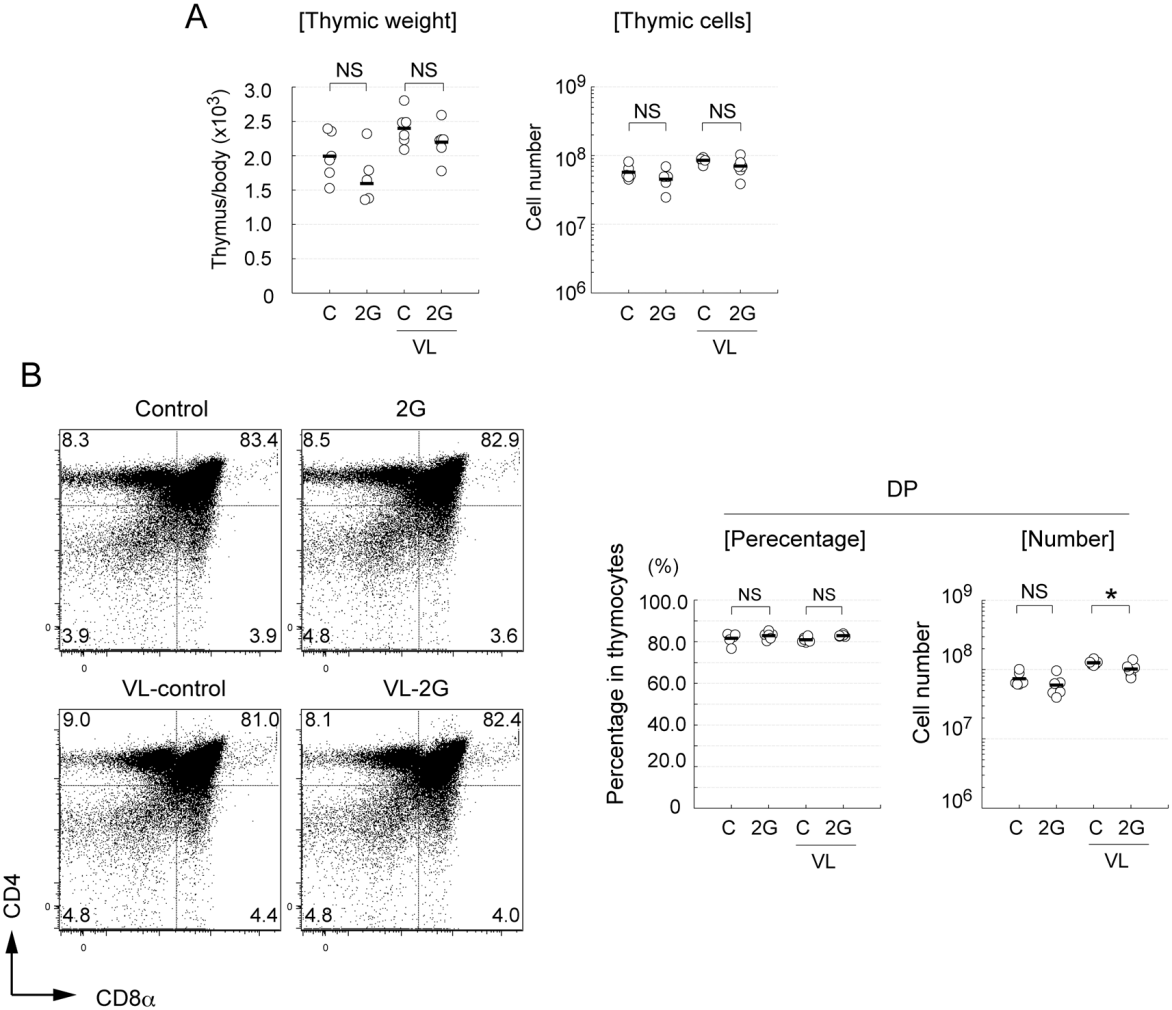
Thymic weight and thymocytes were normal in mice exposed to 2G for 14 days. (A) Ratio of thymic weight to body weight (left) and total thymic cell number (right) of mice exposed to 2 *g* gravity (2G) for 14 day or normal gravity control (C). Vestibular apparatus are surgically disrupted in some groups of mice (labeled as VL). N = 6 each C, C with VL, and 2G with VL groups. N = 5 for 2G. NS indicates that the difference is not significant (Student’s *t*-test). (B) Flow cytometric analysis data of thymocytes in mice exposed to 2G for 14 days. Mice were exposed to 2 *g* gravity (labeled as 2G) for 14 days or left under 1G (control). Vestibular apparatus are surgically disrupted in some groups of mice (VL). Thymocytes were analyzed by staining with CD4 and CD8α antibodies. Numbers in panels indicate percentage of each fraction. Percentage and numbers of CD4- and CD8-double positive thymocytes (DP) are summarized in right figures. N = 6 each C, C with VL, and 2G with VL groups. N = 5 for 2G. The asterisks indicate statistical significance at *P < 0.05 (Student’s *t*-test). NS indicates that the difference is not significant (Student’s *t*-test).

To investigate the effects of longer-term exposure to 2G on the thymus, mice were exposed to 2G for 14 days. In contrast with the 3-day 2G exposure, when mice were exposed to 2G for 14 days, both the thymic weight and cell numbers in 2G-exposed mice were comparable to the control 1G mice (Fig 4A). This is consistent with the previous finding on 2G-dependent thymic involution being recovered during continuous 2G exposure [10]. Flow cytometric analysis confirmed that the percentages of DN, DP, CD4SP, and CD8SP were not altered by the long-term 2G exposure (Fig 4B). Although the DP cell number was slightly reduced by 2G exposure in VL mice, this reduction appears to be minimum (Fig 4B). Thus, these data strongly suggest that the 2G-induced reduction of DP is temporary.

### Hypergravity persistently causes a reduction in mature mTECs and an increment in K5^+^K8^+^ TECs

**Fig 5:**
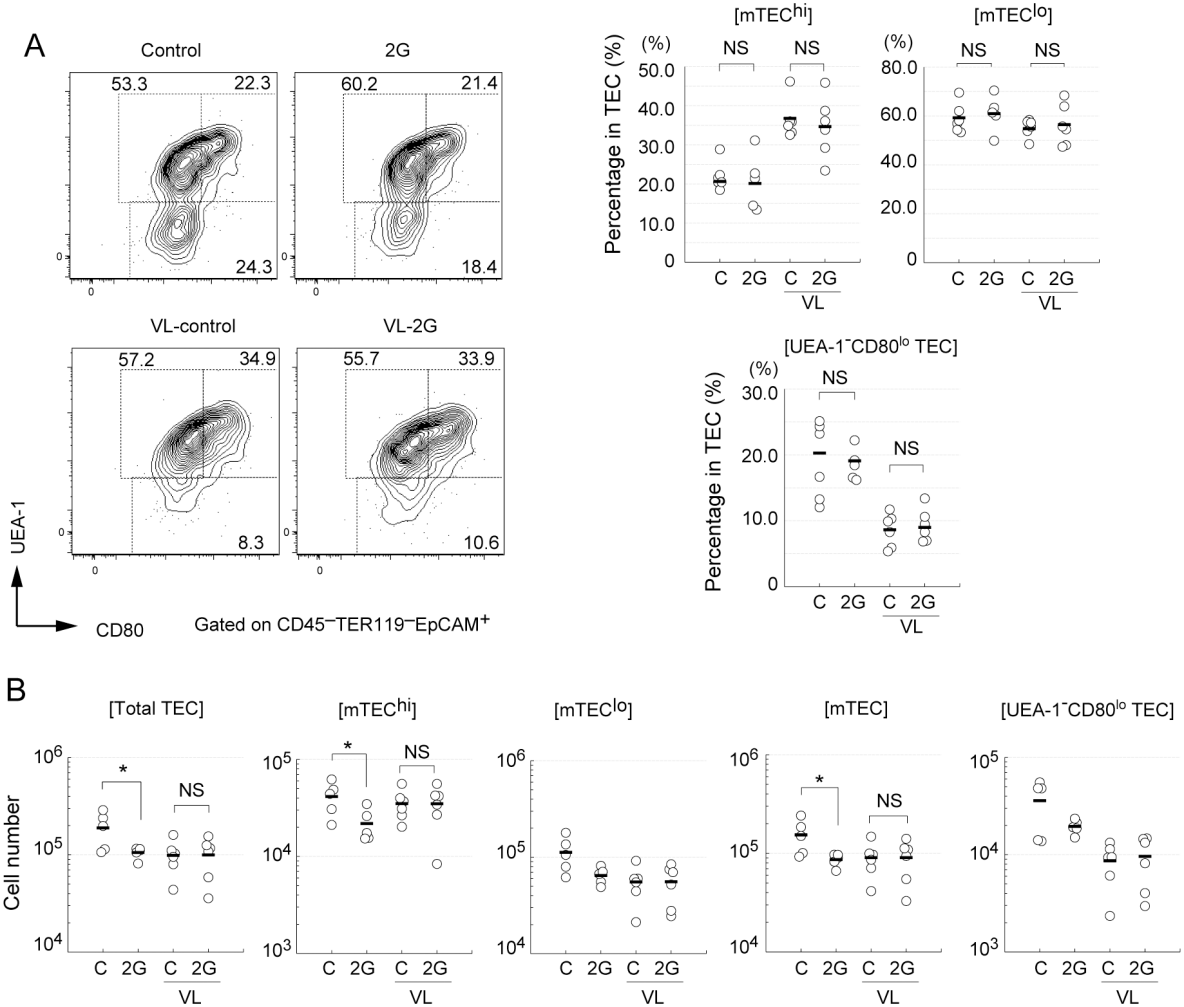
Long-term 2G exposure causes a reduction in cell number of mTECs. (A) Flow cytometric analysis of thymic epithelial cells (TECs) in mice exposed to 2G for 14 days. Mice were exposed to 2 *g* gravity (2G) for 14 days or left under 1G (control). Vestibular apparatus are surgically disrupted in some mice (VL). TECs (CD45^–^TER119^–^ EpCAM^+^) in total thymic cells were analyzed by staining with UEA-1-lectin, an mTEC marker, and CD80 antibody. Numbers in panels indicates percentage of each fraction. The percentages of UEA-1^–^ CD80^lo^ (containing cTECs), UEA-1^+^CD80^high^ (mTEC^hi^), and UEA-1^+^CD80^low^ (mTEC^lo^) cells among thymic stroma cells are summarized in right figures. “C” in graphs indicates 1G control. N = 6 each C, C with VL, and 2G with VL groups. N = 5 for 2G. NS indicates that the difference is not significant (Student’s *t*-test). NS indicates that the difference is not significant (Student’s *t*-test). (B) Cell numbers of total TECs (CD45^–^TER119^–^ EpCAM^+^), UEA-1^–^ CD80^lo^ TECs (containing cTECs), mTECs (UEA-1^+^), mTEC^hi^ (UEA-1^+^CD80^high^), and mTEC^lo^ (UEA-1^+^CD80^low^) in the thymus are summarized in figures. N = 6 each C, C with VL, and 2G with VL groups. N = 5 for 2G. The asterisks indicate statistical significance at *P < 0.05 (Student’s *t*-test). NS indicates that the difference is not significant (Student’s *t*-test).

**Fig 6:**
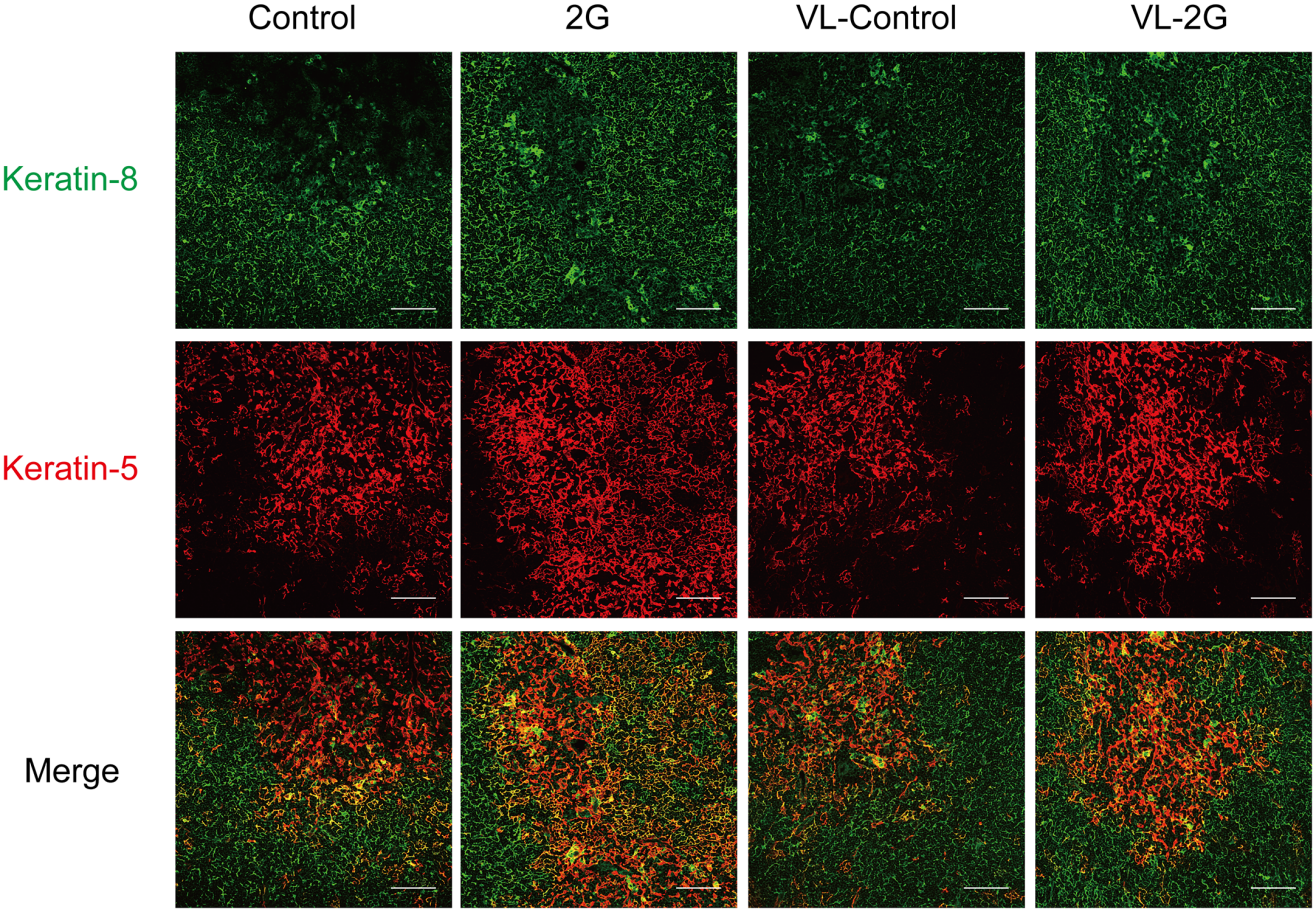
Increment of keratin-5 keratin-8-double positive cells induced by hypergravity is persistent. Immunostaining of thymic section from mice exposed to 2 *g* gravity (2G) for 14 days. Thymic frozen sections were immunostained with a combination of anti-keratin-8 antibodies (upper panels) and anti-keratin-5 (middle panels). Merged images are shown in lower panels. Mice were exposed to 2 *g* gravity (2G) for 3 days or left under 1G (Control). Vestibular apparatus are surgically disrupted in some groups of mice (VL). Data are representatives of 6 independent mice samples (N = 6). Scale bars indicate 100 μm. Data are representatives of 3 independent mice samples (N = 3).

We further investigated the maintenance of TECs in mice exposed to long-term 2G. Flow cytometric analysis showed that the percentages of mTEC and UEA-1^–^CD80^lo^ TEC fractions in total TECs were not practically altered in the thymus exposed to 2G for 14 days (Fig 5A). However, the cell numbers of mTEC^hi^ were significantly reduced by 2G exposure for 14 days (Fig 5B). In addition, although the reduction was not statistically significant, the numbers of other cell fractions were slightly decreased (Fig 5B). In contrast to practically complete recovery of DP, these data suggest that 2G exposure persistently reduces the number of mature mTECs and probably also reduces other TECs. VL itself appears to cause a reduction in the percentage of UEA-1^–^CD80^lo^ TEC fraction and the total TEC numbers for unknown reasons (Fig 5A and 5B). However, it should be noted that 2G-dependency was not evident in TEC of VL mice. An immunohistochemical study suggests that the 2G-dependent increment of K5^+^K8^+^ TECs persistently occurred in the thymus of mice exposed to 2G for 14 days, even though the effect appears milder than that of short-term 2G exposure (Fig 6). Similar to the effect of short-term 2G, the increment of K5^+^K8^+^ by long-term 2G exposure was practically absent in the VL mice (Fig 6).

Overall, these data suggest that long-term exposure to hypergravity causes a persistent impairment of the thymic microenvironment established by TECs. Because mTEC^hi^ was most sensitively affected by hypergravity, we further investigated the expression of genes critical for mTEC development and function in the thymus of 2G-exposed mice.

### Early up-regulation and subsequent down-regulation of Aire and RANK mRNA expression

**Fig 7:**
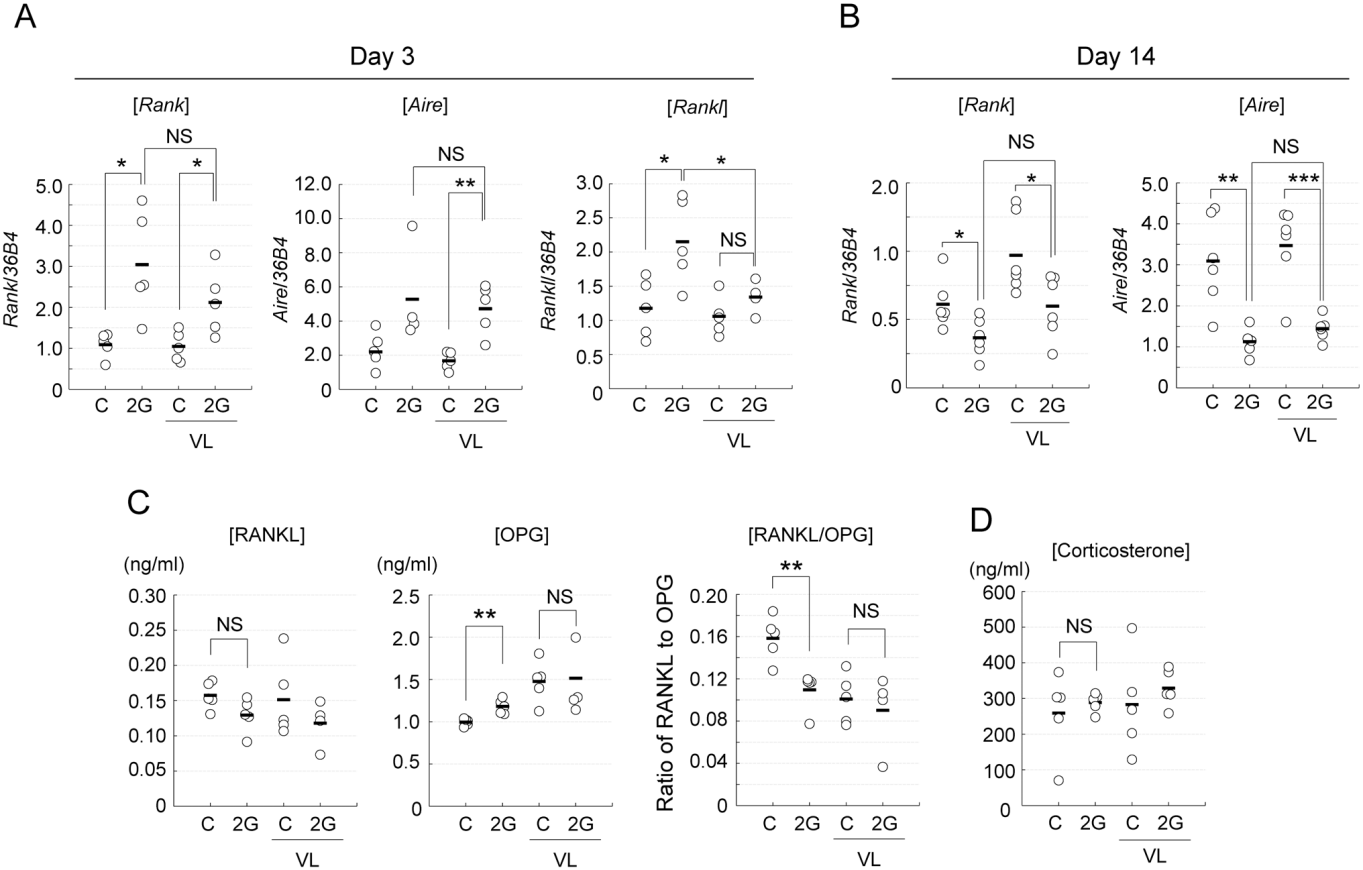
Expressions of mTEC-related molecules in the thymus and plasma of mice exposed to 2G. (A) Expressions of RANK, Aire, and RANKL in the whole thymus of mice exposed to 2G (2G) for 3 days. “C” indicates 1G control. Vestibular apparatus are surgically disrupted in some groups of mice (labeled as VL). Expression of RANK (*Rank*), Aire (*Aire*), and RANKL (*Rankl*) mRNAs was evaluated by qPCR analysis. N = 5 each C, 2G, C with VL, and 2G with VL groups. The asterisks indicate statistical significance at **P < 0.01 and *P < 0.05 (Student’s *t*-test). (B) Expressions of RANK and Aire in the whole thymus of mice exposed to 2G for 14 days. Expression of RANK and Aire mRNAs was evaluated by qPCR analysis. N = 6 each C, C with VL, and 2G with VL groups. N = 5 for 2G. The asterisks indicate statistical significance at *P < 0.05, *P < 0.01, and ***P < 0.001 (Student’s *t*-test). NS indicates that the difference is not significant (Student’s *t*-test). (C) RANKL and OPG protein levels in plasma of mice exposed to 2G for 3 days. Plasma concentration of RANKL (left) and OPG (middle) protein was determined by ELISA. Ratio of RANKL to OPG (RANKL/OPG) was exhibited in the right figure. N = 5 each C, C with VL, and 2G with VL groups. N = 4 for 2G. The asterisks indicate statistical significance at **P < 0.01 (Student’s *t*-test). (D) Corticosterone level in plasma of mice exposed to 2G for 3 days. Concentration of corticosterone was determined by ELISA. N = 6 each C, G, C with VL, and 2G with VL groups. NS indicates that the difference is not significant (Student’s *t*-test).

Previous studies revealed that the development and maintenance of mTECs depend on TNF receptor family signaling [37]. In particular, TNF receptor family RANK signaling plays a critical role in the development of Aire-expressing mTECs [38–40], thereby suppressing the onset of autoimmunity. We investigated the expression level of Aire and RANK in the whole thymus of mice exposed to hypergravity. Exposure to 2G for 3 days significantly increased RANK expression (Fig 7A). Unexpectedly, the up-regulation of RANK expression was observed in the thymus of VL mice. Aire expression was also up-regulated by the 2G exposure in VL mice (Fig 7A) although an increment in the 2G-treated mice did not reach statistical significance. Thus, the 2G-dependent up-regulation of RANK and Aire expression occurs without the vestibular apparatus sensing gravity and therefore can not explained simply by the relative increase in mTEC frequency due to the reduction in DP number, which is dependent on the vestibular apparatus (Fig 1). Surprisingly, in contrast to the 3 day-exposure, 2G exposure for 14 days rather caused a reduction of Aire and RANK expression in the thymus (Fig 5B). Moreover, this 2G-mediated reduction in gene expression is independent of VL. Thus, the reduction of RANK and Aire expression caused by the 14-day 2G exposure also can not explained only by the reduction of mTEC^hi^ number, which is also dependent on the vestibular apparatus (Fig 5). Consequently, these data suggest that 2G exposure caused an early increment and subsequent reduction of mTEC gene expression without any involvement of the vestibular apparatus.

Because the 2G-dependent change in mTEC gene expression cannot be explained simply by the change in thymic cell frequency, we checked other parameters that possibly affect Aire and RANK expression in the thymus. Previous studies suggested that both Aire and RANK expression were up-regulated by the stimulation of thymic stroma cells with RANK ligand (RANKL) [38, 41]. We therefore examined the expression level of RANKL in the thymus after 2G exposure. Indeed, qPCR analysis suggested that 2G exposure enhanced RANKL expression in the thymus (Fig 7A). More importantly, however, was the fact that VL significantly inhibited such increment (Fig 7A), suggesting that the 2G-dependent change in thymic RANKL expression is most likely due to the change of cell composition in the thymus. As result, the 2G-dependent up-regulation of Aire and RANK in the thymus could not be solely explained by the increment of thymic RANKL expression.

RANKL and its natural inhibitor OPG regulate bone homeostasis, and are partially secreted into the blood stream [42]. Therefore, given the fact that gravity change disturbs bone homeostasis [12], the concentrations of RANKL and OPG in the blood stream could possibly be altered, thereby indirectly influencing gene expression in mTECs. We then evaluated the concentrations of plasma RANKL and OPG from mice exposed to 2G for 3 days. Whereas the concentration of plasma RANKL was not changed, the concentration of plasma OPG was significantly increased (Fig 7C). As result, the 2G exposure decreased the ratio of RANKL to OPG (RANKL/OPG; Fig 7C). This suggests that available RANKL in blood stream was rather reduced by 2G. Moreover, the 2G-dependent change in RANKL/OPG was not detected in VL-treated mice (Fig 7C). Therefore, the change in protein concentrations of RANKL or OPG in the blood stream also could not explain the VL-independent increment in thymic RANK and Aire expression by 2G. We also considered the possible effects of corticosterone—a major stress hormone in mice that is secreted in the blood stream—because such a steroid hormone affects the expression of many genes [43]. However, 2G exposure did not practically influence the serum corticosterone level under this condition (Fig 7D), which may be consistent with a previous study [11]. As a result, these blood parameters could not be responsible for the altered expression of thymic RANK and Aire induced by 2G exposure. Therefore, other mechanisms or direct gravity effects could influence the regulatory mechanisms of gene expression in mTECs, which should be explored in a future study.

## Discussion

Our study revealed a 2G-dependent reduction of DP. Such reduction of DP would not be explained by the decrease in TECs because the 3-day exposure to 2G mainly influenced frequency of mTEC^hi^, which contributes to selection of CD4SP and CD8SP rather than DP. Because DP is most sensitive to various stressors and easily undergoes apoptosis, the short-term exposure to 2G might cause apoptosis of DP. Another possibility is that the 2G-exposure enhanced positive selection of DP to maintain number of CD4SP and CD8SP. Such homeostatic maintenance could indirectly cause a reduction in relative number of DP. More detailed analysis on a time-dependent change of thymocyte populations induced by hypergravity may be informative to address this issue in future.

Interestingly, the vestibular lesion remarkably alleviated the reduction of DP. Thus, the sensing of gravity by the vestibular apparatus would be critical for hypergravity-dependent thymic involution. The mechanism linking the sensing of gravity by the vestibular apparatus to the reduction of DP thymocytes remains unknown. Although corticosterone is a possible candidate for mediating gravity stress, we failed to detect an increment of corticosterone in 2G exposure for 3 days. However, our data can not rule out the possibility that an acute and transient increment of corticosterone occurs at an earlier time point, thereby inducing the temporary reduction of DP thymocytes because a previous study using rats suggested a vestibular apparatus-dependent increment of corticosterone by exposure to hypergravity [44]. A detailed analysis of the corticosterone level at different time points will be informative in the future. Another possibility is that a reduction in food intake caused by gravity change [45] provokes acute fasting and thereby affects the thymocytes population. However, it should be noted that the 2G-dependent reduction in cell number was significant only in DP, whereas starvation reportedly caused reductions in all types of thymocytes including CD4SP, CD8SP, and DN [46]. Thus, starvation may not fully explain the selective reduction of DP thymocytes induced by 2G exposure.

Our data indicated that 2G exposure results in the increment of K5^+^K8^+^ TECs. Previous studies suggested that K5^+^K8^+^ TECs contain TEC progenitors that differentiate into mature TECs [30, 31, 47, 48]. Moreover, irradiation and dexamethasone treatment (which mimic the action of stress hormones) promote the increment of Ki67-positive K5^+^K8^+^ TECs [36], implying that the recovery of TECs after removing stressors is dependent on the proliferation of these immature TECs. Our data suggest that 2G exposure initiates a similar mechanism of TEC recovery. A recent study suggested the involvement of IL-22 of which expression is dependent on IL23-expressing dendritic cells (DCs) during TEC recovery from irradiation [49]. In that study, the reduction of DP thymocytes induces IL-23 expression in DCs. It would be interesting to investigate whether the IL22-dependent system is active in the thymus of mice exposed to 2G.

Because a recent study suggested that K5^+^K8^+^ population could represent mTEC precursor populations [32], the hypergravity might cause an increment of K5^+^K8^+^ mTEC precursors rather than TEC precursors. Interestingly, we found the preferential reduction of mTEC^hi^ population by 2G exposure. Thus, it is also possible that an impaired differentiation of mTECs by hypergravity leads to accumulation of mTEC precursors expressing both keratin-5 and keratin-8.

We found that 2G exposure changed the expression of Aire and RANK, which are critical for the function and development of mTECs. Intriguingly, we found that these genes were up-regulated at an early time point and then later down-regulated. The mechanism of this dynamic change currently remains unknown. However, it should be noted that these 2G-dependent alterations of the gene expression level are independent of the vestibular apparatus. Furthermore, this gene expression change does not apparently correlate with the corticosterone level and ratio of RANKL to OPG in the blood stream. Therefore, other gravity sensing systems (e.g. shift of body fluid) or a direct gravity effect on mTECs might be responsible for the changes of these gene expressions in mTECs. More detailed and comprehensive gene expression analysis is thus necessary for uncovering the mechanism to regulate this effect of hypergravity.

Importantly, the Aire expression was reduced in the thymus of mice exposed to 2G for 14 days, whereas CD4SP and CD8SP develop normally in the thymus. Therefore, given that Aire is critical for preventing autoimmunity, the 2G-treated thymus may possibly generate self-reactive T cells with higher frequency. Such a situation could increase the risk of autoimmunity.

Overall, we obtained some new findings about the hypergravity-dependent effects on the maintenance of thymocytes, mature TECs, and mTEC gene expression. These data might contribute to understanding the effects of gravity change on immune systems in spaceflight and living in the future.

## Materials and Methods

### Ethics statement

Animals used in the present study were maintained in accordance with the “Guiding Principles for Care and Use of Animals in the Field of Physiological Science” set by the Physiological Society of Japan. The experiments were approved by the Animal Research Committees of Gifu University, the Animal Research Committees of the Aerospace Exploration Agency, and Committee for Animal Experiments of the Institute of Medical Science, University of Tokyo (approved number H13-26).

### Mice and Antibody

C57BL/6 mice were purchased from Chubu Kagaku Shizai (Nagoya, Japan). All the mice were handled in accordance with the Guidelines for Animal Experiments, Gifu University (Gifu, Japan). Mice are sacrificed by cervical dislocation. The APC-Cy7-conjugated CD4, PE-Cy-7-conjugated CD8 antibodies, PE-conjugated CD80 antibody, the purified anti-mouse CD16/32, the APC-Cy7-conjugated rat anti-mouse CD45, APC-Cy7-conjugated TER-119, and PE-conjugated anti-mouse EpCAM (G8.8) were obtained from Biolegend (San Diego, CA). The rabbit anti-mouse keratin-5 antibody was purchased from Covance (Berkeley, CA). The biotinylated anti-mouse keratin-8 antibody was from PROGEN (Heidelberg, Germany). The biotinylated UEA-1 was obtained from VECTOR Laboratories (Burlingame, CA). Further, 7-aminoactinomycin D was purchased from Wako (Tokyo, Japan).

### Lesion of vestibular apparatus and induction of hypergravity

Six-week-old mice were anesthetized with an isoflurane (Escain, Pfizer, Tokyo, Japan) inhalation via a face mask and the VL (lesion of vestibular apparatus) surgery was performed through an external auditory meatus approach. After removal of the tympanic membrane, malleus, incus, and stapes, labyrinthine fluid was aspirated. A #20 or #25 file (Mani, Utsunomiya, Japan) was inserted into the oval window and surrounding bone was resected, and then electrical cautery was applied through the file. The success of the VL was confirmed by observing the swimming behavior of the mice after they were gently placed in a small tub filled with warm water. Mice with complete lesions were unable to determine the direction in which they had to swim to reach the water surface, and continued to turn around under the water. When we observed this behavior, the swim test was immediately ended, and mice were rescued. Swim test was performed once for each mouse. It takes a few second to perform one swim test. When Penicillin G potassium (3000 U/kg, Meiji Seika Pharma, Tokyo, Japan) and buprenorphine (3 μg/kg, Lepetan, Otsuka, Tokyo, Japan) were administered subcutaneously prior to returning animals to their home cages. Mice were placed in a custom-made aluminum cage (35 x 25 x 17 cm), which was placed inside the rotating box of the centrifuge immediately after surgery to allow the rats to become accustomed to the environment of the experimental room. The temperature in the experimental room was maintained at 24°C. Two weeks after the surgery, gravitational stress in the dorsoventral direction was applied to the mouse in the prone posture by centrifugation using a custom-made gondola-type rotating box (Shimadzu, Kyoto, Japan) for indicated times. A gravity sensor (MTS-050, Mitec, Hiroshima, Japan) was placed on the cage floor to measure gravity.

### Flow cytometric analysis of thymic cells

Total thymic cells were prepared by digestion in RPMI 1640 medium containing Liberase (Roche) and DNase I (Sigma). For thymocytes analysis, single cell suspensions from the thymus were prepared mechanically. Before staining, cells were preincubated with anti-Fcγ III/II receptor (2.4G2) (BD Pharmingen) to block Fc receptors. Cells were stained with antibodies in PBS containing 2% FBS. Dead cells were excluded from the analysis by 7-aminoactinomycin D (Wako) staining. Stained cells were analyzed with a fluorescence-activated cell sorter (FACS; Canto II; BD Bioscience).

### Immnouhistochemistry

Thymus were embedded in OCT compound (Sakura Finetek, Tokyo, Japan) and frozen in liquid nitrogen. Cryostat sections (6 μm thick) were fixed by ice-cold acetone and incubated with primary antibody solution for 1 h at room temperature. The slides were subsequently incubated with secondary antibody for 40 min at room temperature. Confocal color images were obtained using an Olympus FV1000D at 20× or 60x (for Fig 6F) magnification and Fluoview software (Olympus).

### Quantitative RT-PCR

Total RNA was extracted using TRIZOL (Invitrogen) and was subjected to random-primed reverse transcription using the Primescript II 1^st^ strand cDNA synthesis kit (TAKARA Bio.). Quantitative real-time RT-PCR was performed using an ABI PRISM 7300 Sequence Detection System (Applied Biosystems) and SYBR Green Master Mix (Roche or TOYOBO). Primer sequence used are for RANK-fwd; GCTGGCTACCACTGGAACTC, RANK-rev; GTGCAGTTGGTCCAAGGTTT, Aire-fwd; GGT TCTGTTGGACTCTGCCCTG, Aire-rev; TGTGCCACGACGGAGGTGAG, RANKL-fwd; TGTACTTTCGAGCGCAGATG, and RANKL-rev; AGGCTTGTTTCATCCTCCTG

### ELISA

Plasma corticosterone levels were determined by Corticosterone EIA kit (Cayman Chemical Company, Ann Arbor, MI) according to the manufacturer’s instructions. Plasma samples were assayed at a 1:600 dilution. Absorbance at 405nm was measured using an iMark microplate reader (BioRad). The plasma levels of RANKL and OPG were measured using Mouse TRANCE/RANK L/TNFSF11 Quantikine ELISA Kit and Mouse Osteoprotegerin/TNFRSF11B Quantikine ELISA Kit (R&D Systems, Minneapolis, Minnesota) according to the manufacture’s instructions. The plasma RANKL/OPG ratio was shown by a ratio of plasma RANKL level to the plasma OPG level.

## Supporting Information

S1 Fig Analysis of thymocytes in mice exposed to 2G for 3 days. Percentages of CD4^+^CD8^-^ (CD4SP), CD4^-^CD8^+^ (CD8SP), CD4^-^CD8^-^ (DN) cells are summarized in graphs. Mice were exposed to 2 *g* gravity (2G) for 3 days or left under 1G (control). Vestibular apparatus are surgically disrupted in some groups of mice (VL). N = 5 each C, 2G, C with VL, and 2G with VL groups. The asterisks indicate statistical significance at ***P < 0.001 (Student’s *t*-test). NS indicates that the difference is not significant (Student’s *t*-test).
(TIF)

S2 Fig TEC analysis in Fig 2 was repeated by using different mouse groups. (A) Mice were exposed to 2 *g* gravity (2G) for 3 days or left under 1G (control). Vestibular apparatus are surgically disrupted in some mice (VL). TECs (CD45^–^TER119^–^ EpCAM^+^) in total thymic cells were analyzed by staining with UEA-1-lectin, an mTEC marker, and CD80 antibody. Numbers in panels indicates percentage of each fraction. The percentages of UEA-1^–^ CD80^lo^ (containing cTECs), UEA-1^+^CD80^high^ (mTEC^hi^), and UEA-1^+^CD80^low^ (mTEC^lo^) cells among thymic stroma cells in the thymus are summarized in right figures. N = 5 each C and 2G, N = 4 each C with VL, and 2G with VL groups. The asterisks indicate statistical significance at **P < 0.01 (Student’s *t*-test). (B) Cell numbers of total TECs (CD45^–^TER119^–^ EpCAM^+^), UEA-1^–^ CD80^lo^ TECs (containing cTECs), mTECs (UEA-1^+^), mTEC^hi^ (UEA-1^+^CD80^high^), and mTEC^lo^ (UEA-1^+^CD80^low^) in the thymus are summarized in figures. N = 5 each C, 2G, C with VL, and 2G with VL groups.
(TIF)

S3 Fig Analysis of thymocytes in mice exposed to 2G for 14 days. Percentages and cell number of CD4^+^CD8^-^ (CD4SP), CD4^-^CD8^+^ (CD8SP), CD4^-^CD8^-^ (DN) cells are summarized in graphs. Mice were exposed to 2 *g* gravity (2G) for 14 days or left under 1G (control). Vestibular apparatus are surgically disrupted in some groups of mice (VL). N = 6 each C, C with VL, and 2G with VL groups. N = 5 for 2G. The asterisks indicate statistical significance at *P < 0.05 (Student’s *t*-test).
(TIF)

## Abbreviations

Aire: autoimmune regulator
CD4SP: CD4^+^CD8^-^ T cells
CD8SP: CD4^-^CD8^+^ T cells
cTECs: cortical thymic epithelial cells
DN: CD4^–^CD8^–^ thymocytes
DP: CD4^+^CD8^+^ thymocytes
K5^+^K8^+^: keratin-5 and keratin-8 double-positive
mTECs: medullary thymic epithelial cells
RANK: receptor activator of NF-κB
TECs: thymic epithelial cells
VL: lesion of vestibular apparatus of the inner ear
