# Supplementary figures and images for: Hypergravity Provokes a Temporary Reduction in CD4+CD8+ Thymocyte Number and a Persistent Decrease in Medullary Thymic Epithelial Cell Frequency in Mice
Source: PLoS One. 2015 Oct 29;10(10):e0141650. doi: 10.1371/journal.pone.0141650 (PMC4626100; doi:10.1371/journal.pone.0141650)

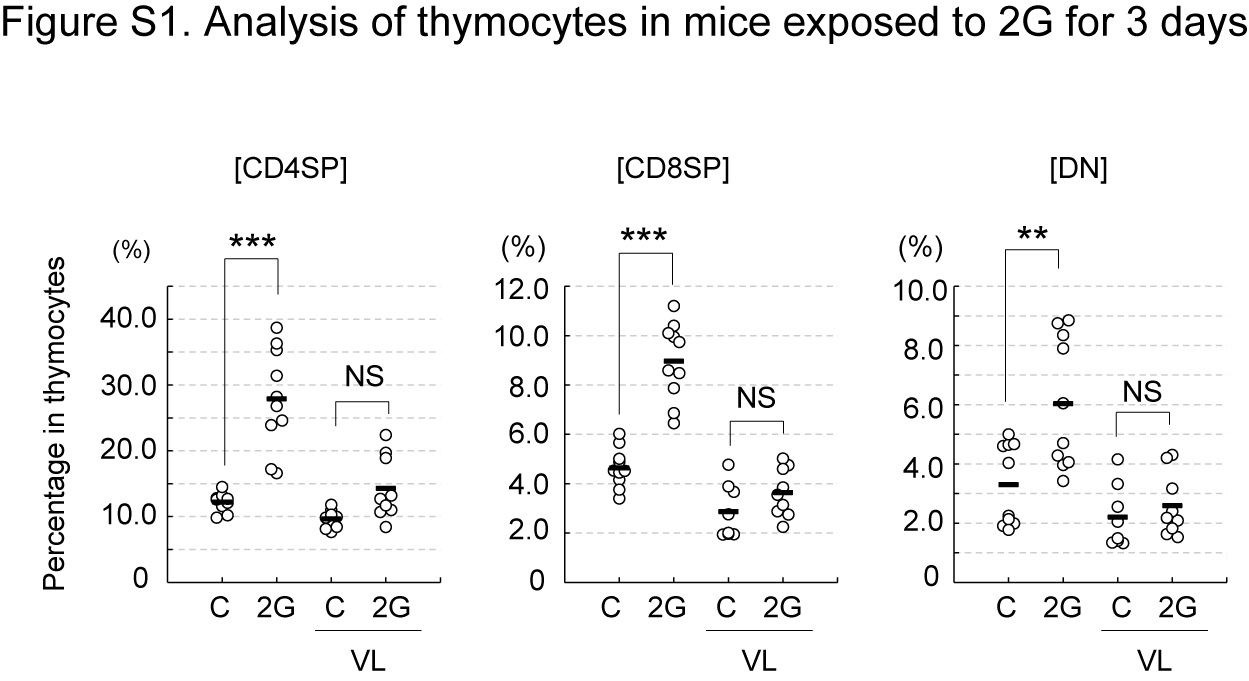

Supplement: S1 Fig — Percentages of CD4+CD8- (CD4SP), CD4-CD8+ (CD8SP), CD4-CD8- (DN) cells are summarized in graphs. Mice were exposed to 2 g gravity (2G) for 3 days or left under 1G (control). Vestibular apparatus are surgically disrupted in some groups of mice (VL). N = 5 each C, 2G, C with VL, and 2G with VL groups. The asterisks indicate statistical significance at ***P < 0.001 (Student’s t-test). NS indicates that the difference is not significant (Student’s t-test). (TIF) [file pone.0141650.s001.tif]

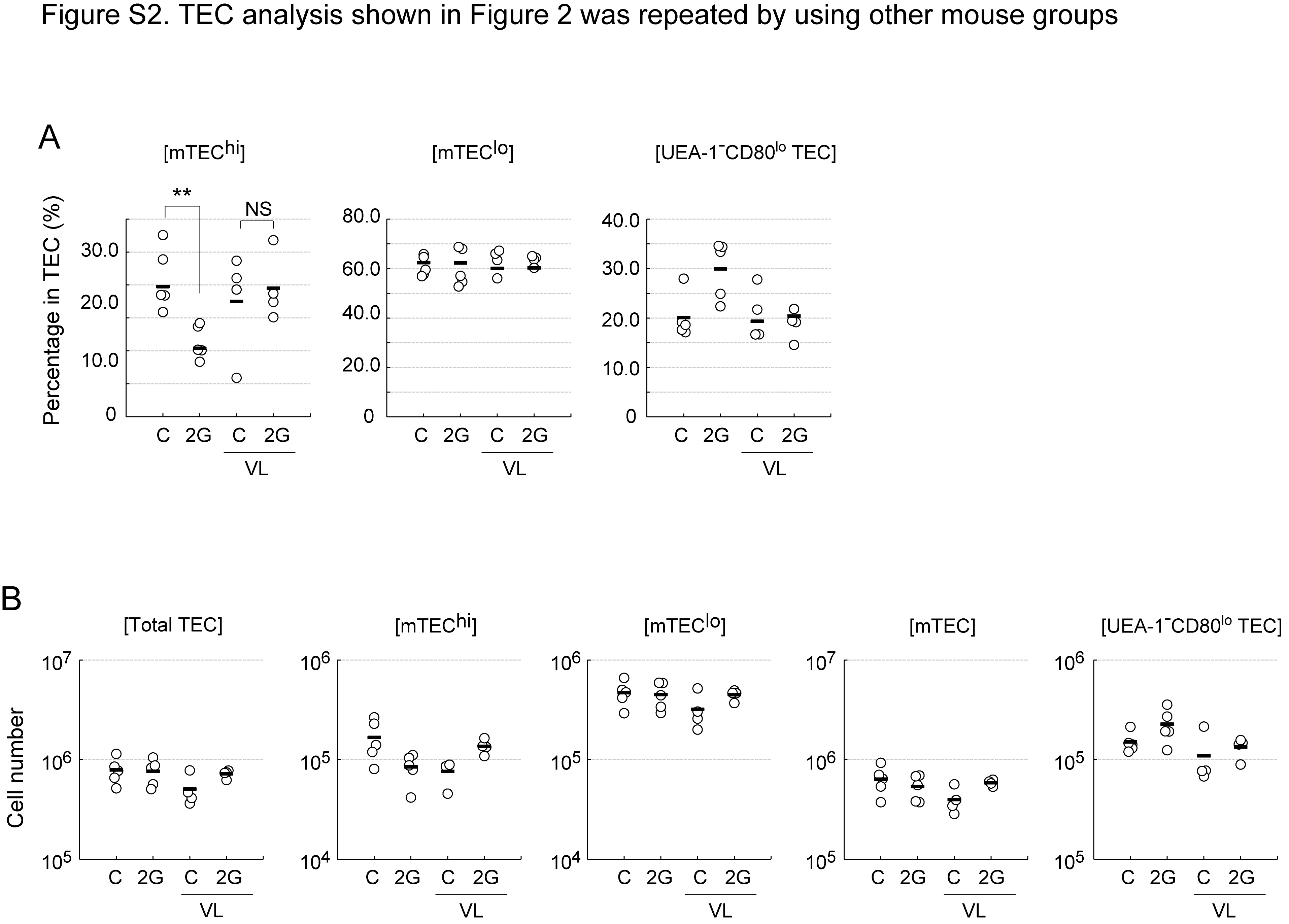

Supplement: S2 Fig — (A) Mice were exposed to 2 g gravity (2G) for 3 days or left under 1G (control). Vestibular apparatus are surgically disrupted in some mice (VL). TECs (CD45–TER119– EpCAM+) in total thymic cells were analyzed by staining with UEA-1-lectin, an mTEC marker, and CD80 antibody. Numbers in panels indicates percentage of each fraction. The percentages of UEA-1– CD80lo (containing cTECs), UEA-1+CD80high (mTEChi), and UEA-1+CD80low (mTEClo) cells among thymic stroma cells in the thymus are summarized in right figures. N = 5 each C and 2G, N = 4 each C with VL, and 2G with VL groups. The asterisks indicate statistical significance at **P < 0.01 (Student’s t-test). (B) Cell numbers of total TECs (CD45–TER119– EpCAM+), UEA-1– CD80lo TECs (containing cTECs), mTECs (UEA-1+), mTEChi (UEA-1+CD80high), and mTEClo (UEA-1+CD80low) in the thymus are summarized in figures. N = 5 each C, 2G, C with VL, and 2G with VL groups. (TIF) [file pone.0141650.s002.tif]

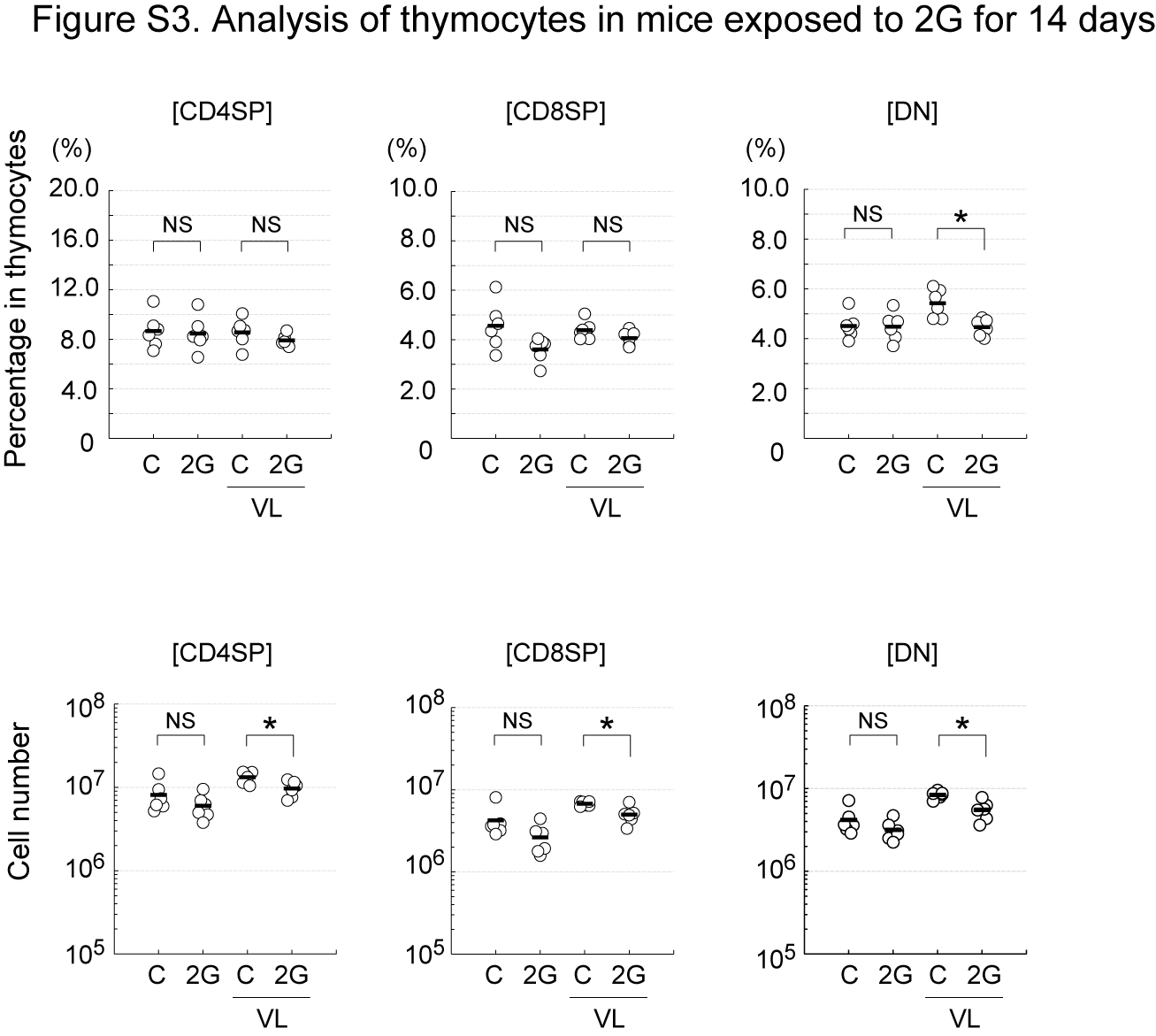

Supplement: S3 Fig — Percentages and cell number of CD4+CD8- (CD4SP), CD4-CD8+ (CD8SP), CD4-CD8- (DN) cells are summarized in graphs. Mice were exposed to 2 g gravity (2G) for 14 days or left under 1G (control). Vestibular apparatus are surgically disrupted in some groups of mice (VL). N = 6 each C, C with VL, and 2G with VL groups. N = 5 for 2G. The asterisks indicate statistical significance at *P < 0.05 (Student’s t-test). (TIF) [file pone.0141650.s003.tif]
